# Virtual noncontrast images reveal gouty tophi in contrast-enhanced dual-energy CT: a phantom study

**DOI:** 10.1186/s41747-024-00466-w

**Published:** 2024-06-12

**Authors:** Karim Khayata, Torsten Diekhoff, Jürgen Mews, Sydney Schmolke, Maximilian Kotlyarov

**Affiliations:** 1 Department of Radiology, Charité - Universitätsmedizin Berlin, Campus Mitte, Humboldt-Universität Zu Berlin, Freie Universität Berlin, Charitéplatz 1, Berlin, 10117 Germany; 2 Canon Medical Systems, Europe BV, Zoetermeer, The Netherlands

**Keywords:** Contrast media, Gout, Phantoms (imaging), Tomography (x-ray computed), Uric acid

## Abstract

**Background:**

Dual-energy computed tomography (DECT) is useful for detecting gouty tophi. While iodinated contrast media (ICM) might enhance the detection of monosodium urate crystals (MSU), higher iodine concentrations hamper their detection. Calculating virtual noncontrast (VNC) images might improve the detection of enhancing tophi. The aim of this study was to evaluate MSU detection with VNC images from DECT acquisitions in phantoms, compared against the results with standard DECT reconstructions.

**Methods:**

A grid-like and a biophantom with 25 suspensions containing different concentrations of ICM (0 to 2%) and MSU (0 to 50%) were scanned with sequential single-source DECT using an ascending order of tube current time product at 80 kVp (16.5–220 mAs) and 135 kVp (2.75–19.25 mAs). VNC images were equivalently reconstructed at 80 and 135 kVp. Two-material decomposition analysis for MSU detection was applied for the VNC and conventional CT images. MSU detection and attenuation values were compared in both modalities.

**Results:**

For 0, 0.25, 0.5, 1, and 2% ICM, the average detection indices (DIs) for all MSU concentrations (35–50%) with VNC postprocessing were respectively 25.2, 36.6, 30.9, 38.9, and 45.8% for the grid phantom scans and 11.7, 9.4, 5.5, 24.0, and 25.0% for the porcine phantom scans. In the conventional CT image group, the average DIs were respectively 35.4, 54.3, 45.4, 1.0, and 0.0% for the grid phantom and 19.4, 17.9, 3.0, 0.0, and 0.0% for the porcine phantom scans.

**Conclusions:**

VNC effectively reduces the suppression of information caused by high concentrations of ICM, thereby improving the detection of MSU.

**Relevance statement:**

Contrast-enhanced DECT alone may suffice for diagnosing gout without a native acquisition.

**Key points:**

• Highly concentrated contrast media hinders monosodium urate crystal detection in CT imaging

• Virtual noncontrast imaging redetects monosodium urate crystals in high-iodinated contrast media concentrations.

• Contrast-enhanced DECT alone may suffice for diagnosing gout without a native acquisition.

**Graphical Abstract:**

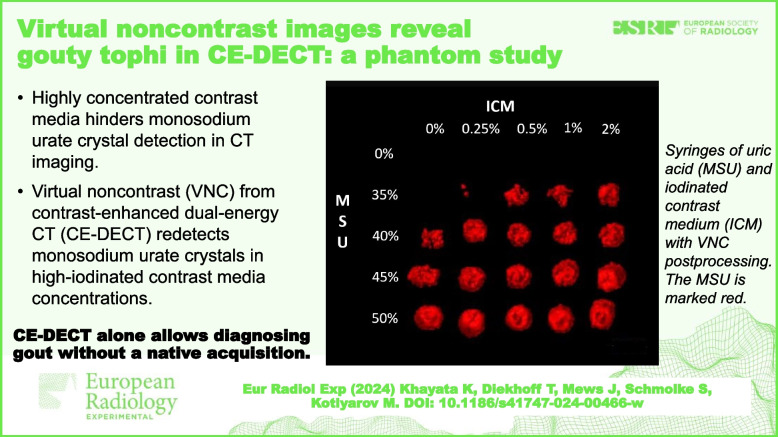

## Background

Gout is a disease characterized by a granulomatous inflammatory response to the presence of monosodium urate (MSU) crystals in the joints, bones, and soft tissues with the formation of the so-called tophi [1]. The tophus contains cellular, humoral, and crystal components causing the clinical symptoms of gout, which typically presents itself as an acute monoarticular flare in the lower extremities. However, atypical and chronic forms of gout, such as involvement of the axial skeleton or polyarticular manifestations, can mimic other diseases like rheumatoid or psoriatic arthritis and may lead to wrong therapy decisions [2, 3].

Dual-energy computed tomography (DECT) has proven its efficacy in the detection of MSU using the different attenuation properties of tissues in different voltages [4]. Moreover, contrast-enhanced multimodal DECT has gained relevance in recent years, allowing the evaluation of periarticular soft tissue inflammation in addition to erosions, bone marrow edema, and other lesions [5, 6]. A single contrast-enhanced DECT scan in patients with an unclear arthritis etiology can thus provide objective evidence for active inflammation or rule out differential diagnoses. A recent phantom study of gouty arthritis has shown that DECT images acquired after administration of iodinated contrast medium (ICM) can improve the detection of tophi with a lower density that would remain undetected in unenhanced CT images [7]. At the same time, however, high ICM almost completely hampered tophus detection. Therefore, tophi with a strong contrast uptake may be obscured.

Virtual noncontrast (VNC) imaging is a postprocessing method for DECT scans. VNC images are generated by subtracting iodine maps from the contrast-enhanced images. This results in a set of native images that can be compared to an acquisition without administering iodine contrast to the patient. If successfully applied, this subtraction technique could potentially enable the identification of monosodium urate (MSU) despite high ICM concentrations.

The aim of our study was to investigate MSU detection in VNC images generated from contrast-enhanced DECT datasets.

## Methods

Due to the phantom nature of the study, approval by the institutional review board was not necessary.

### Phantom setup and scan protocol

**Fig. 1:**
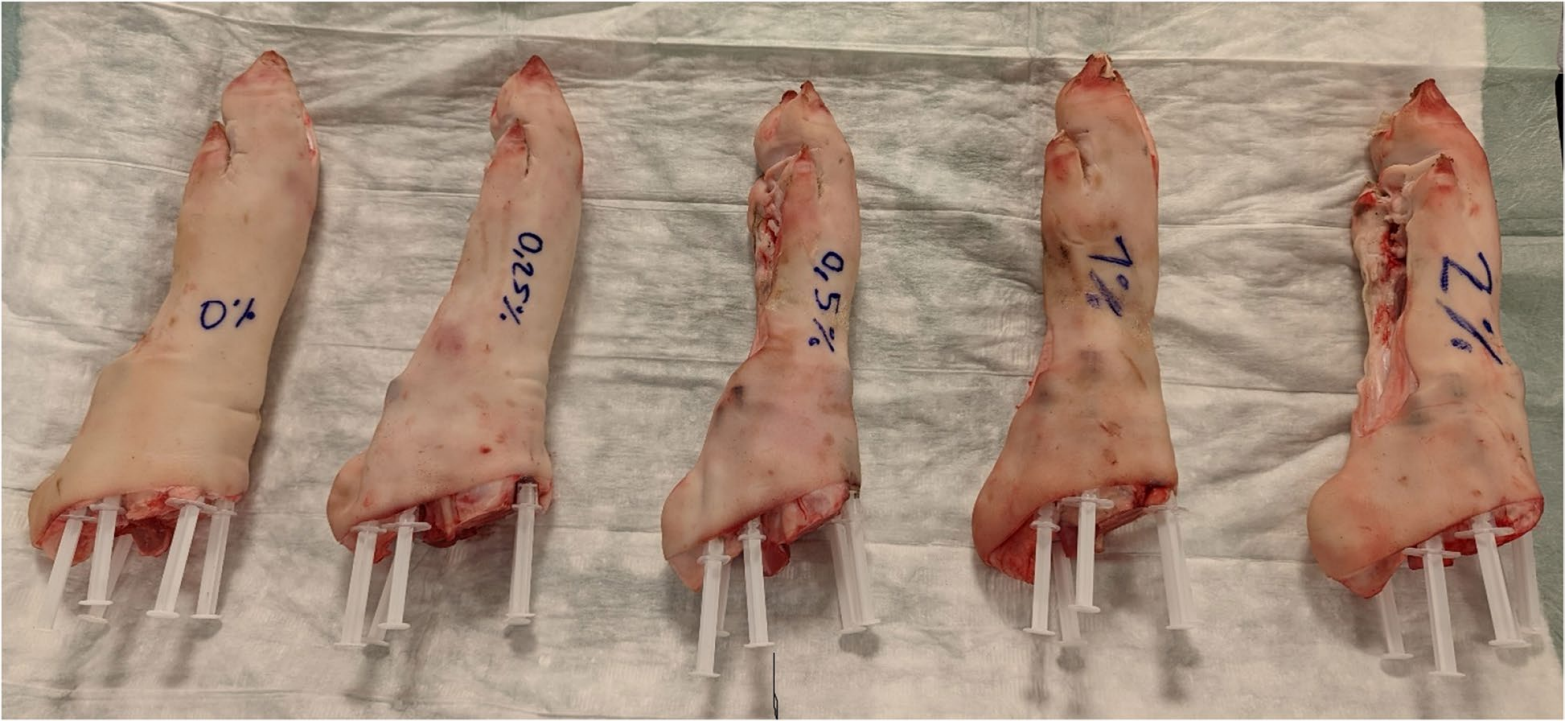
Porcine legs with respectively five inserted syringes containing the same ICM concertation

To simulate different concentrations of MSU *in vivo*, we used two established phantom models with 25 suspensions containing 3 components in various concentrations: MSU (linear formula, *C*5*H*3*N*4*O*3*Na*; molecular weight, 190.09; *Z*_eff_, 7.7; Sigma Aldrich, St. Louis, MO, USA), ultrasound gel, and ICM containing 370 mg iodine/mL (Ultravist 370, iopromide; linear formula, *C*18*H*24*I*3*N*3*O*8; molecular weight, 791.1; *Z*_eff_, 41.4; Bayer Vital GmbH, Leverkusen, Germany). Five sets of suspensions containing MSU concentrations of 0%, 35%, 40%, 45%, and 50% mixed with varying ICM concentrations of 0%, 0.25%, 0.5%, 1%, and 2% (0.925, 1.85, 3.7, and 7.4 mg/mL of iodine, respectively) were prepared. The five MSU concentrations were selected based on previous work [7–9]. The suspensions were filled in 25 individual syringes. MSU masses were measured and documented. For the first phantom, the syringes were placed in a grid-like structure immersed in water. For the second phantom, a biophantom, five freshly prepared porcine forelegs with intact cutaneous, subcutaneous, muscular, and bone structures were bought from the slaughterhouse to mimic gouty tophi in realistic surroundings. In each foreleg, five syringes containing the same ICM concentration, but different MSU concentrations, were placed in the tissue surrounding the joint (Fig. 1).

All phantoms were scanned in a 320-row scanner (Aquilion ONE Vision Edition; Canon Medical Systems, Otawara, Japan) with the “dual-energy volume scan” mode with a total number of 10 acquisitions per phantom using a single-source technique with 2 sequential scans at 80 kVp and 135 kVp with 16-cm *z*-axis coverage without table movement and a rotation time of 0.275 s. The 10 acquisitions had an ascending order of tube currents ranging from 60 to 800 mA at 80 kVp and 10 to 140 mA at 135 kVp. All scans were reconstructed using adaptive iterative dose reduction at a strong iteration level (AIDR3D_strong_) with a medium soft tissue kernel without beam hardening compensation (FC13), a 0.5-mm slice thickness, and a 0.25-mm reconstruction interval. The tube current–time product, CT dose index, and dose-length product were documented, and the estimated effective dose was calculated using a conversion coefficient for upper extremities of 0.0008 $\frac{{\text{mSv}}}{{\text{mGy}}\times {\text{cm}}}$ [10, 11].

### Image postprocessing

Three-material decomposition was used to calculate iodine maps from the original images, resulting in two VNC images equivalent to 80 and 135 kVp. All 60 phantom acquisitions were reconstructed using AIDR3D_strong_ image reconstructions (10 grid phantom scans and 50 porcine biophantom scans). The VNC images were generated using a proprietary clinical software package (version 6; Canon Medical Systems). Standard software settings by the vendor were used for calculation: water, 0/0 HU; MSU, 100/107 HU for low and high kVp with a gradient of 0.55 for iodine.

The calculated sets of two VNC images at 80 and 135 keV were processed in the same way as conventional DECT scans using a two-material decomposition analysis algorithm (Dual-Energy Composition Analysis, version 6.0, Canon Medical Systems) for the detection of uric acid using clinical standard settings established in previous studies [7–12].

The 60 phantom acquisitions without any prior subtraction were processed as conventional CT images with the same two-material decomposition analysis algorithm, thereby creating our reference group.

### Quantitative analysis

The MSU volumes detected using two-material decomposition analysis and MSU masses estimated using an established method were recorded for both VNC images and conventional CT images [12]. False-positive detections within the phantom but outside of the syringes were classified as artifacts and quantified by their volume. Due to the variability in absolute MSU content within the syringes, we established a detection index (DI) to enhance the comparability of results.

$$Detection\;index\left[\mathit\%\right]\mathit=\left(\frac{detected\mathit\;MSU\mathit\;mass\mathit\;\left[\textit{mg}\right]}{real\mathit\;MSU\mathit\;mass\mathit\;\left[\textit{mg}\right]}\right)\times100$$

The resulting DIs from the VNC images were compared with the results of the datasets of the conventional CT images, described in detail in a previous study [7].

Attenuation values of the syringes were measured in the grid-like and pig foreleg phantom. Consistency was maintained by employing the same layer, location, and size for the region of interest in both VNC and conventional CT images.

### Statistical analysis

Statistical significance was measured by using a paired *t*-test for intergroup comparability after VNC usage. Statistical analysis and visualization of data were carried out using Microsoft Excel (2016, Microsoft Corporation, Redmond, WA, USA) and Jupyter Notebook (2022, Anaconda Inc., Austin, TX, USA) using the pandas, matplotlib, scipy, and seaborn libraries.

## Results

### Phantom setup and scan protocol

Phantom setup and imaging were executed successfully. All acquisition and dose parameters are summarized in Table 1.

**Table 1:** Acquisition parameters

I (mA) at 80 kVp	I (mA) at 135 kVp	Q (mAs) at 80 kVp	Q (mAs) at 135 kVp	CTDI (mGy)	DLP (mGy × cm)	EED (mSv)
60	10	16.5	2.75	1	16.1	0.013
90	15	24.75	4.125	1.5	24.1	0.019
110	20	30.25	5.5	1.9	30.5	0.024
170	30	46.75	8.25	2.9	46.6	0.037
230	40	63.25	11	3.9	62.6	0.05
290	50	79.75	13.75	4.9	78.7	0.063
400	70	110	19.25	6.8	109.2	0.087
510	90	140.25	24.75	8.7	139.7	0.112
630	110	173.25	30.25	10.7	171.8	0.137
800	140	220	38.5	13.6	218.4	0.175

### Post CT image processing

**Fig. 2:**
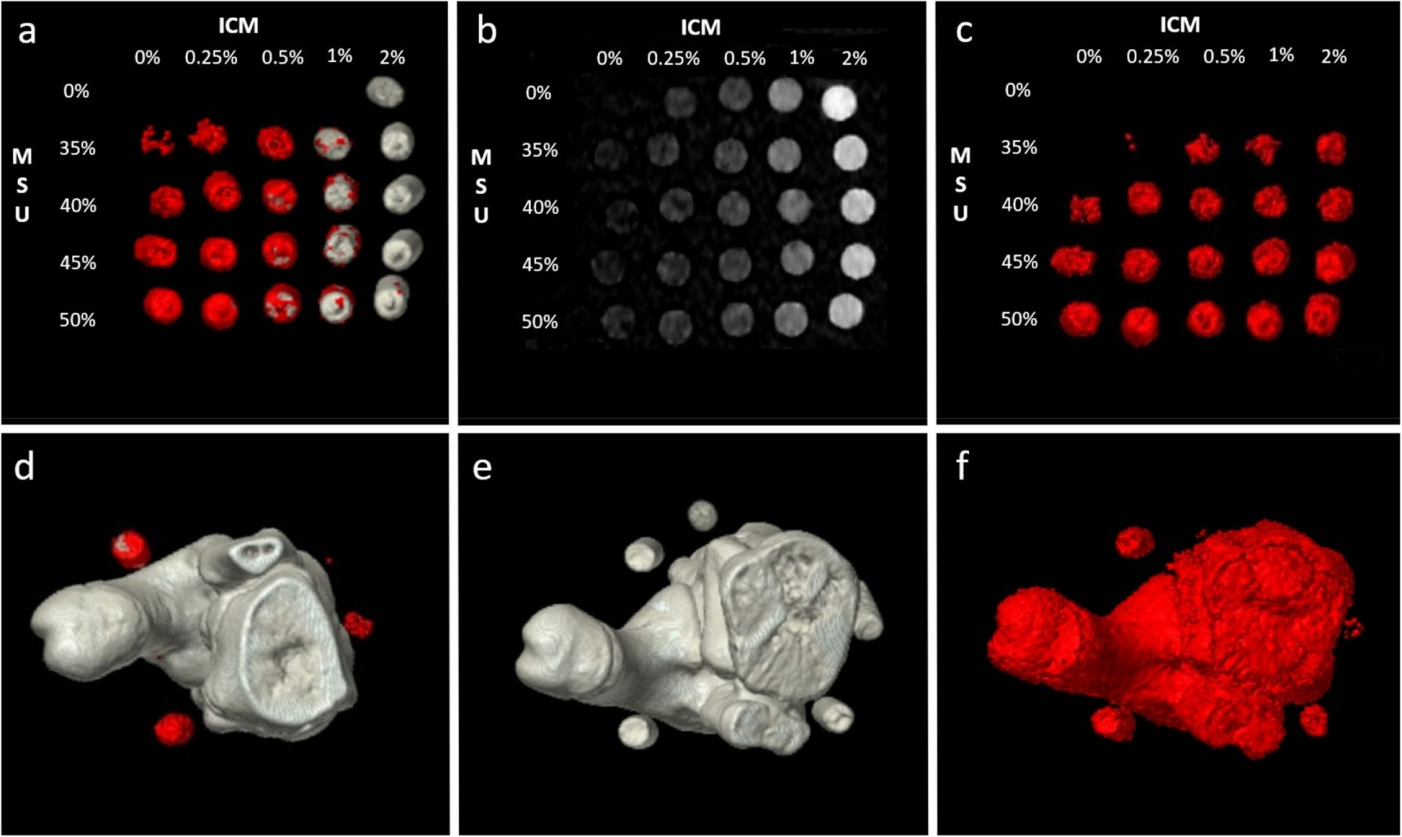
Upper row (grid phantom): The syringes are ordered by their concentration of iodinated contrast medium (ICM) in ascending order and monosodium urate (MSU) in descending order. **a** MSU map without virtual-non-contrast (VNC), highlighting detected MSU coded in red. **b** Iodine map (ICM coded in gray). **c** MSU map with VNC postprocessing. Lower row (porcine phantom): The syringes are placed around the elbow joint in ascending clockwise order. **d** MSU map of a phantom without ICM showing MSU detection. **e** Phantom with 2% ICM completely hampering the MSU detection. **f** VNC postprocessing enables MSU detection in the same phantom with 2% ICM. False-positive MSU detection in bone structure is observed

All datasets were successfully postprocessed. False-positive MSU artifacts inside the bone compartment were excluded from the analysis. For images of the postprocessing steps, please see Fig. 2.

### Quantitative analysis

**Fig. 3:**
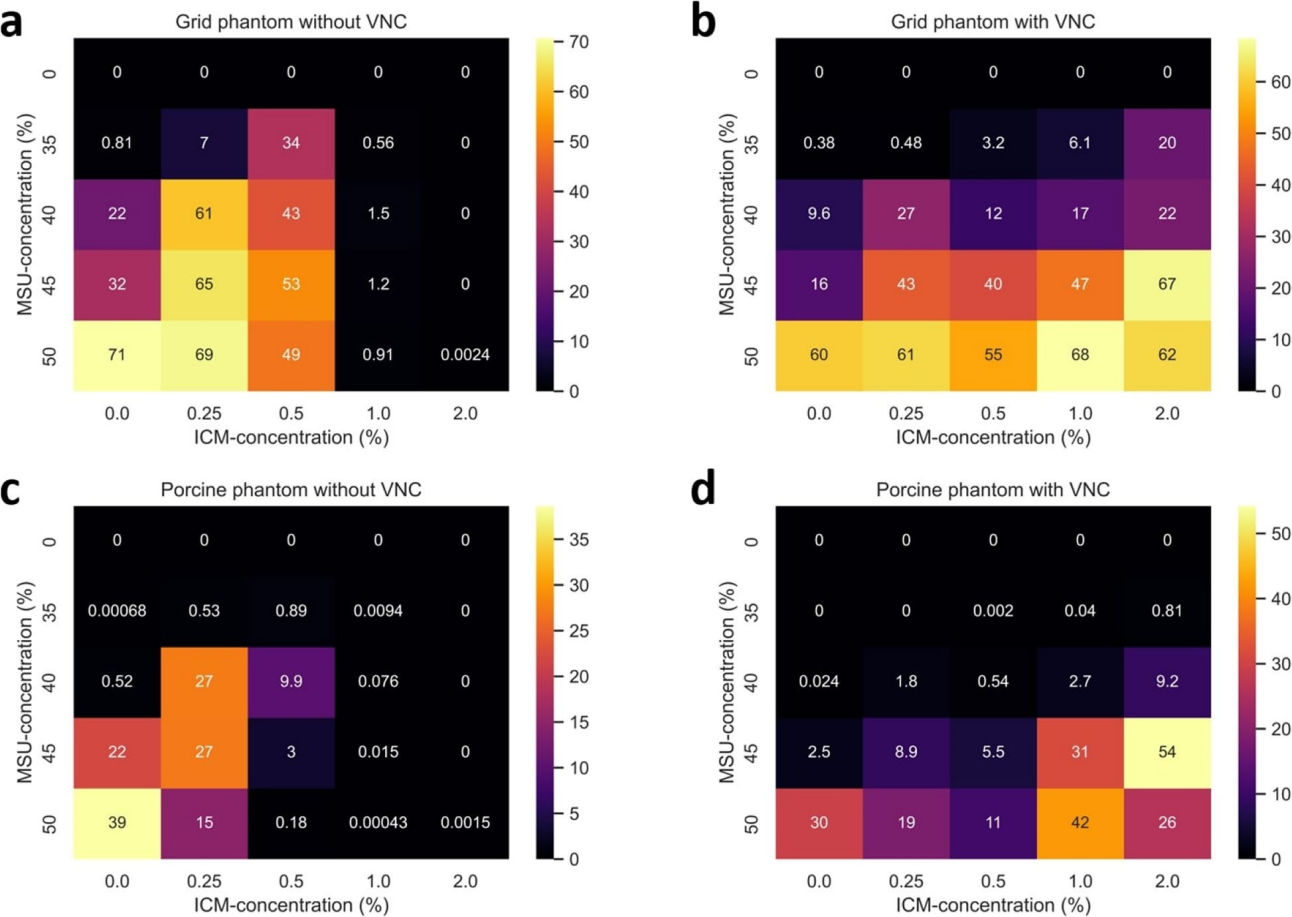
Heatmaps of detection indices. Results are shown for the grid phantom (**a**, **b**) and the porcine phantom (**c**, **d**) with the use of virtual noncontrast (VNC) and conventional CT images. The *x*-axis in each of the heatmaps represents the concentration of iodinated contrast medium (ICM), while the *y*-axis represents the uric acid (MSU) concentration. The heatmap is ordered similarly to our grid phantom array. Each of the squares contains the value of the mean detection index (DI) of every syringe and is color-coded according to the color bar on the right side. Both in the grid-like and in the biophantom, VNC imaging enables MSU detection even in the presence of higher ICM concentrations (1% and 2%), at which no MSU is detected without VNC imaging

With VNC postprocessing, the average DIs for all MSU concentrations (35–50%) and tube currents were 25.2, 36.6, 30.9, 38.9, and 45.8% respectively for 0, 0.25, 0.5, 1, and 2% ICM for the grid phantom and 11.7, 9.4, 5.5, 24.0, and 25.0% for the porcine biophantom. In conventional CT images, the average DIs were respectively 35.4, 54.3, 45.4, 1.0, and 0.0% for the grid phantom and 19.4, 17.9, 3.0, 0.0, and 0.0% for the porcine biophantom. The results are presented in detail in Fig. 3.

The DIs measured in the grid phantom images were significantly higher than those of the porcine biophantom across all ICM concentrations investigated. While DIs varied significantly with the concentrations of MSU and ICM present in the syringes and the tube currents used, no trend was discernible.

**Fig. 4:**
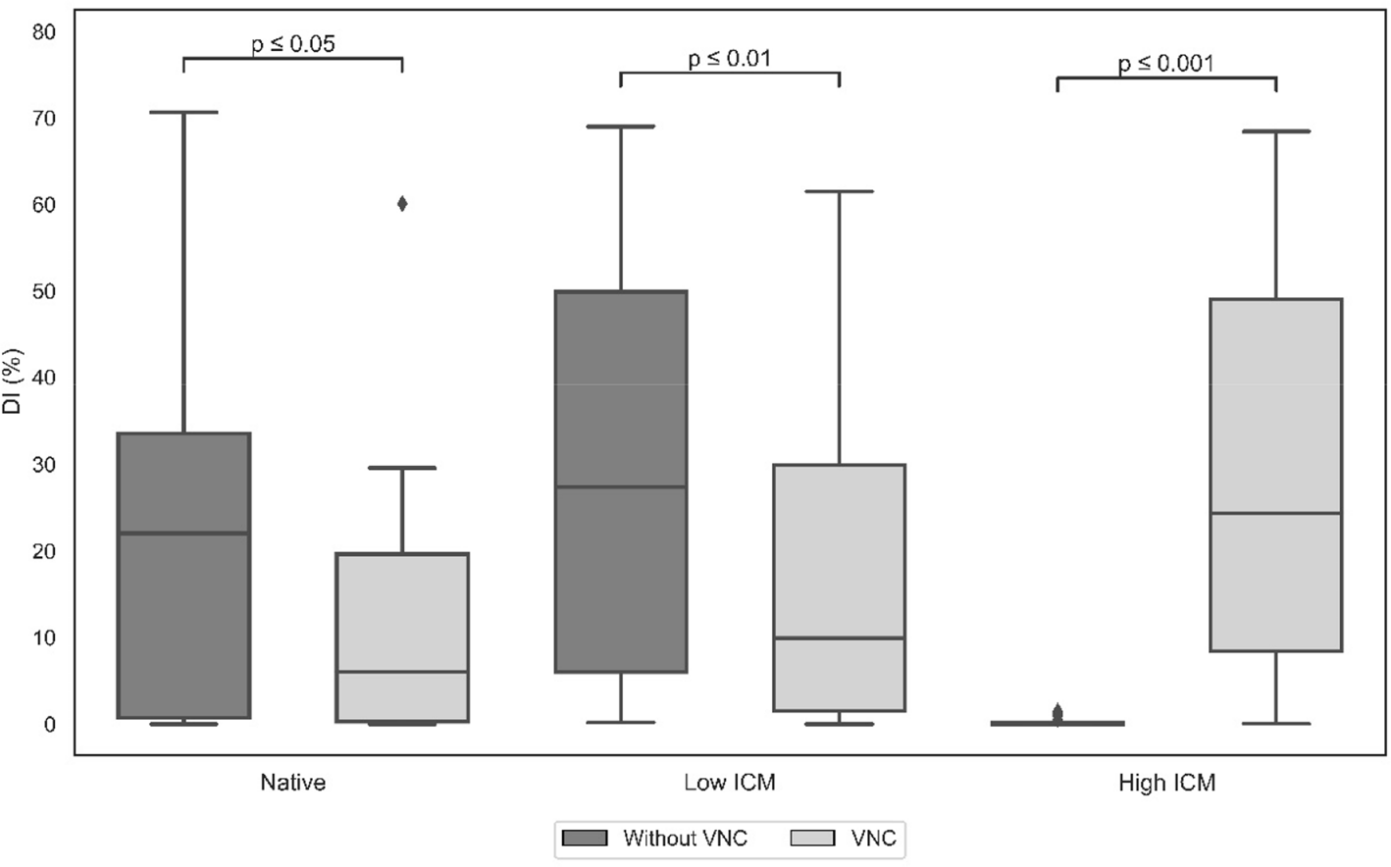
Detection index for all iodinated contrast medium (ICM) concentrations. Boxplot diagram with different phantom setups (*x*-axis) and their respective DI (*y*-axis): contrast-enhanced images without the use of virtual noncontrast (VNC) imaging have higher DI in low ICM concentrations (0.25% and 0.5%), while DI are reduced to a value of zero in high ICM concentrations (1% and 2%). VNC images show lower DI in noncontrast and low IC concentrations (0.25% and 0.5%), while reaching significantly higher results in higher ICM concentrations (1% and 2%); *p*-values are shown above the compared groups

In syringes with higher ICM concentrations (1% and 2%), the use of VNC images significantly improved MSU detection to a rate similar to that achieved for conventional CT images without ICM. The VNC images showed lower DIs in syringes without ICM or with lower ICM concentrations (0.25 and 0.5%) in comparison with conventional CT images (*p* = 0.015 and *p* = 0.008, respectively). For comparisons, see Fig. 4.

Syringes not containing MSU never showed false-positive MSU detection. There was also no false-positive MSU detection in the surrounding connective and soft tissue components in the porcine legs.

In conventional CT images, attenuation values in syringes with 50% MSU in the grid phantom showed averages of 263.2, 279.2, 289.7, 364.6, and 446.9 HU in 80 kVp and 257.5, 267.9, 270.7, 324.5, and 369.7 HU in 135 kVp for 0.0, 0.25, 0.5, 1.0, and 2% ICM, respectively. With VNC imaging, syringes with 50% MSU in the grid phantom showed average attenuations of 218.9, 221.7, 214.0, 238.9, and 238.6 HU in 80 kVp equivalent images and 233.2, 236.3, 229.0, 255.4, and 255.2 HU in 135 kVp equivalent images for 0.0, 0.25, 0.5, 1.0, and 2% ICM, respectively.

**Fig. 5:**
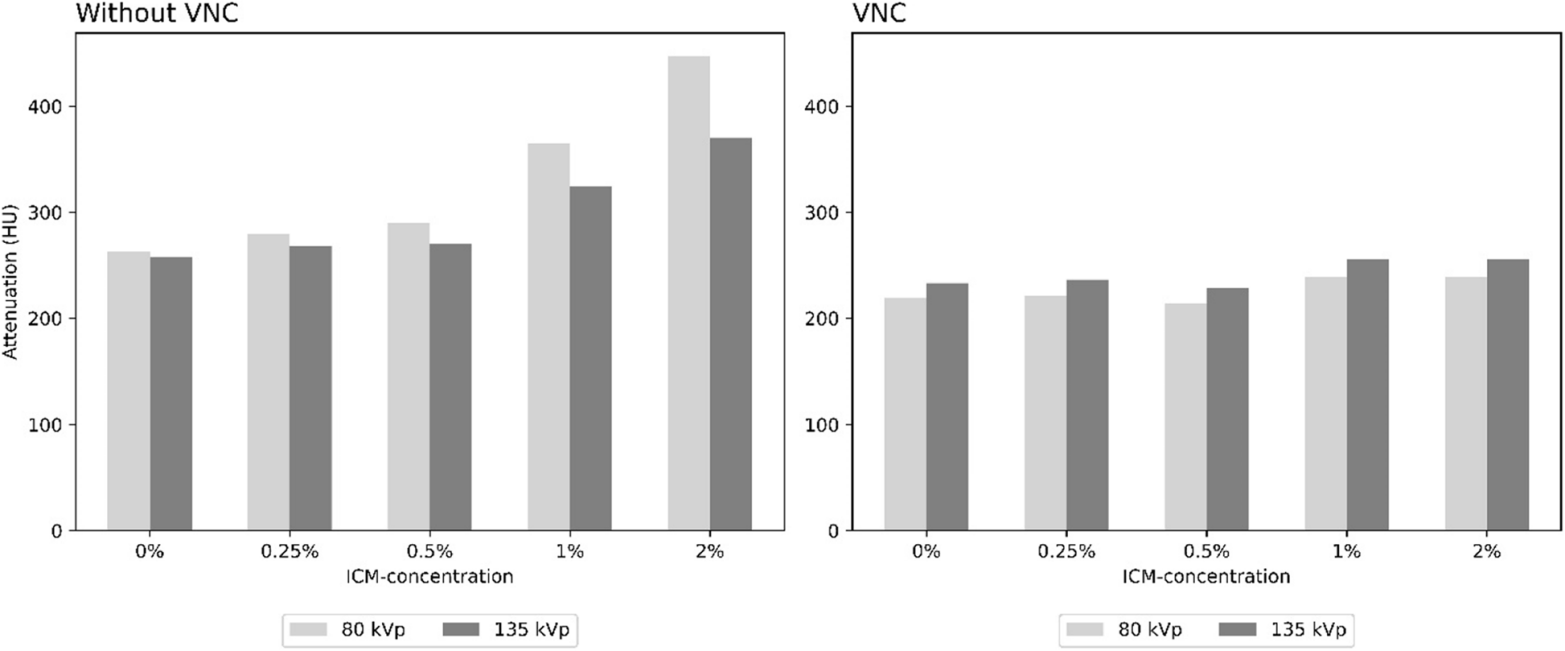
Attenuation values for 50% monosodium urate (MSU) concentration. Exemplary visual representation of attenuation values in 50% MSU syringes in the raster phantom under varying tube voltages (80 and 135 kVp). Attenuation values in enhanced images were increasingly higher at 80 kV than at 135 kV, converging toward the dual-energy gradient of iodine (≈ 0.55) as the ICM concentration increased. In virtual noncontrast (VNC) imaging, the dual-energy gradient (≈ 1.05) becomes optimized for MSU detection

Attenuation measurements showed lower values in VNC images and revealed an increasing difference between VNC and conventional CT images as the concentration of ICM increased for both phantoms and all tube voltages investigated. In all ICM concentrations, VNC successfully altered the attenuation values to match a dual-energy gradient optimized for MSU detection. For an explanatory example, see Fig. 5.

## Discussion

This phantom study shows that VNC imaging enables the detection of MSU crystals even in the presence of high ICM concentrations, thereby resolving the hampering effect of iodine on MSU detection described before [7].

We assessed MSU volumes in phantoms using a two-material decomposition analysis. Subsequently, we estimated MSU masses through an established method that considers local attenuation levels. Given the acknowledged limited sensitivity of DECT for detecting non-tophaceous gout, as reported in other studies [12–14], owing to its reliance on local MSU concentrations and subthreshold CT attenuation, we employed the DI to objectively substantiate our findings.

The DIs found in our study for syringes with higher ICM concentrations in VNC images showed comparable magnitudes to those of scans with low or no ICM without VNC postprocessing. However, syringes with low or no ICM showed lower DIs in VNC imaging than the same syringes in images without VNC postprocessing.

Two-material decomposition differentiates between tissues by taking advantage of their different attenuation properties at different voltages. MSU is an organic purine derivative with a low effective atomic number of *Z*_eff_ 7.7, similar to that of soft tissues. In DECT imaging, MSU is distinguished from soft tissues by setting a certain threshold value. This threshold can be modified to improve either the sensitivity or specificity of detection [7].

Adding an iodinated contrast medium, which has a high *Z*_eff_ of 41.4, can therefore have two distinct effects on a voxel: (i) it raises the density of the voxel and (ii) it increases the *Z*_eff_ of the tissue within the voxel, thus moving the dual-energy index toward that of iodine.

These mechanisms explain the positive results of VNC postprocessing on detection for syringes with higher ICM concentrations as subtraction of iodine reduces the *Z*_eff_ and thus enables proper characterization. On the other hand, poorer MSU sensitivity with VNC in samples with a low iodine concentration is easily explained by the previously described beneficial effect of ICM on tophus detection: ICM raises the attenuation values above the detection threshold without grossly affecting the dual-energy index, thus enhancing the characterization of MSU. Removing iodine negates this effect. Finally, the lower detection in VNC images of MSU samples without ICM needs attention. We used standard clinical settings for postprocessing, which include soft tissue and fat as basis materials to calculate the VNC images. However, MSU and other components typically found in the joints are not considered in this formula. This is also evident through the impact of VNC imaging on calcium detection. Our application of VNC imaging leads to a partial elimination of the ability to differentiate between lesions containing calcium and those containing MSU (Fig. 2f), as discussed by other authors [15]. These factors might result in an unwanted influence of the VNC postprocessing that might be eliminated or optimized in further research. Therefore, we recommend MSU analysis with both VNC and conventional CT images, when suspecting gouty tophi in a contrast-enhanced DECT.

Interestingly, a very similar issue exists in the imaging of urinary calculi, which consist of uric acid. Unenhanced CT is the reference standard for the detection of stones in the urinary tract because they can be obscured by ICM in the renal parenchyma or collecting system. Several phantoms and *in vivo* studies have shown that VNC postprocessing resolves the hampering effect of ICM, consistent with our findings [16–19].

In recent years, DECT has evolved into a powerful multiparametric tool in arthritis imaging, enabling the detection of erosions, bone marrow edema, and MSU depositions [6, 20, 21]. With the application of ICM and the use of iodine maps, it even allows the detection of increased perfusion in synovitis and other musculoskeletal inflammatory lesions [22, 23].

Our data show that, depending on the enhancement level, MSU detection in contrast-enhanced DECT can vary dramatically, without an actual change in a tissue MSU content. In clinical practice, the local ICM concentration is influenced by the perfusion and inflammatory activity in a tophus. This poses a possible restriction in the quantification of MSU volume in serial examinations over the course of the disease as well as considering the given amount of iodine during the contrast administration. These dependencies might preclude objective monitoring of the response to urate-lowering therapy in comparison with the sole use of unenhanced DECT, as discussed by other authors [24].

Our study has several limitations. Firstly, despite our meticulous preparation of the syringes, we cannot entirely rule out the possibility of inhomogeneous distribution of the syringe contents. Secondly, it is important to note that our results may not be readily applicable to other DECT systems, since different vendors offer varying hardware and software configurations, which could yield different outcomes.

Additionally, our use of VNC postprocessing was not optimized for periarticular imaging and was primarily designed to differentiate three materials which might affect the depiction of other structures such as calcium-containing tissue. Furthermore, it is essential to acknowledge that our study was conducted as a phantom study with clinical parameters while lacking a patient cohort to investigate clinical relevance. This limitation leaves open the question of whether contrast uptake of tophi in actual patients would reach the high levels of ICM analyzed in our study. More research with patient data is needed to establish clinical significance.

In summary, this study demonstrates that VNC imaging successfully reduces the hampering effects caused by high ICM concentrations in the detection of MSU crystals by DECT. While the full clinical implications of these findings require further investigation, it appears feasible to opportunistically screen for gout by analyzing contrast-enhanced DECT scans with and without VNC postprocessing, especially when highly vascularized lesions are present. Nevertheless, it is advisable to perform an unenhanced scan when gouty arthritis is suspected to ensure comparability of follow-up examinations.

## Data Availability

The datasets used and/or analyzed during the current study are available from the corresponding author upon reasonable request.

## Abbreviations

CT: Computed tomography
DECT: Dual-energy CT
DI: Detection index
ICM: Iodinated contrast medium
MSU: Monosodium urate
VNC: Virtual noncontrast
